# A Comprehensive Multidisciplinary Approach to Diagnosing Chronic Inflammatory Bowel Diseases: Integration of Clinical, Endoscopic, and Imaging Modalities

**DOI:** 10.3390/diagnostics14141530

**Published:** 2024-07-16

**Authors:** Clelia Cicerone, Ferdinando D’Amico, Mariangela Allocca, Alessandra Zilli, Tommaso Lorenzo Parigi, Silvio Danese, Federica Furfaro

**Affiliations:** 1 Department of Gastroenterology and Endoscopy, IRCCS San Raffaele Hospital, 20132 Milan, Italy; cicerone.clelia@hsr.it (C.C.); damico.ferdinando@hsr.it (F.D.); allocca.mariangela@hsr.it (M.A.); zilli.alessandra@hsr.it (A.Z.); parigi.tommaso@hsr.it (T.L.P.); sdanese@hotmail.com (S.D.); 2 Department of Gastroenterology and Endoscopy, Vita-Salute San Raffaele University, 20132 Milan, Italy

**Keywords:** inflammatory bowel diseases, histology, endoscopy, intestinal ultrasound, biomarkers, pre-clinical inflammation, advanced imaging techniques

## Abstract

Chronic inflammatory bowel diseases, such as Crohn’s disease and ulcerative colitis, present diagnostic challenges due to their complex and heterogeneous nature. While histology remains fundamental for accurate diagnosis, a multidisciplinary approach incorporating clinical, endoscopic, and imaging modalities is increasingly recognized as essential for comprehensive evaluation. This article delves into the importance of integrating various diagnostic techniques in the assessment of IBD. Colonoscopy and histology, with its ability to directly visualize the intestinal mucosa, play a central role in the diagnostic process. However, histological analysis alone may not suffice, necessitating the inclusion of advanced imaging techniques, such as magnetic resonance enterography (MRE), computed tomography enterography (CTE), and intestinal ultrasound (IUS). These techniques provide valuable insights into the disease’s extent, severity, and complications, and should be used in conjunction with biochemical parameters. These modalities complement traditional endoscopic and histological findings, offering a more holistic understanding of the disease process. A multidisciplinary approach that incorporates clinical, endoscopic, histological, serological, and imaging assessments enables clinicians to achieve a more accurate and timely diagnosis of IBD. Moreover, this integrated approach facilitates personalized treatment strategies tailored to individual patient needs, ultimately improving clinical outcomes and quality of life for those affected by chronic inflammatory bowel diseases.

## 1. Introduction

**Figure 1:**
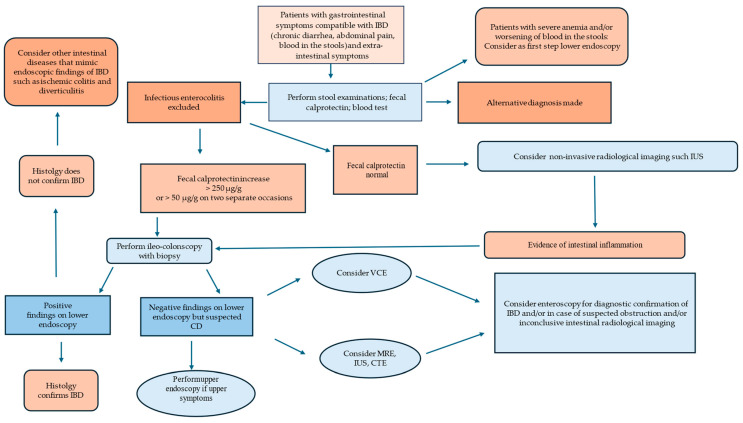
Diagnostic algorithm for patients with suspected IBD. Inflammatory bowel disease: IBD, Crohn’s disease: CD, video capsule endoscopy: VCE, intestinal ultrasound: IUS, magnetic resonance imaging: MRE, CTE: computed tomography enterography.

Inflammatory bowel diseases (IBDs), including Crohn’s disease (CD) and ulcerative colitis (UC), are chronic immune-mediated conditions, characterized by relapsing and remitting intestinal inflammation. UC primarily affects the colon, causing inflammation limited to the mucosal layer. CD can affect any part of the digestive tract, from the mouth to the anus, and often involves deeper layers beyond the mucosa (transmural inflammation) [1]. According to the European Crohn’s and Colitis Organization (ECCO) guidelines, the diagnosis of CD and UC is based on a combination of clinical, biochemical, stool, endoscopic, and histological investigations [2]. While the precise etiology of IBD remains elusive, it is believed that a combination of genetic predisposition, environmental factors, alterations in the gut microbiome, and dysregulated immune responses contribute to gastrointestinal inflammation [3]. Historically, IBD was most common in North America and Europe, primarily affecting people of Western European descent. By the late 20th century, however, this view was shattered, and IBD is now recognized as a global health concern. Population studies indicate a notable increase in the prevalence and incidence of IBD, with a 46% rise over a two-decade period, affecting approximately 0.2% of the European population, totaling 2.5–3 million individuals [4,5,6]. The incidence of CD in Europe ranged between 0.4 and 22.8 per 100,000 person-years, and the incidence of UC was generally higher, ranging between 2.4 and 44.0 per 100,000 person-years [5]. Various risk factors have been associated with IBD. Meta-analyses have demonstrated an increased risk of CD development (RR 1.61) post-appendectomy and a decreased risk of UC [7,8]. Additionally, antibiotic exposure in infancy has been linked to a heightened risk of CD (OR 1.74; 95% CI: 1.35–2.23) [9]. Further factors, including smoking, urban residence, tonsillectomy, oral contraceptive use, soft drink consumption, vitamin D deficiency, and non–Helicobacter pylori-like enterohepatic Helicobacter species, have been identified as increasing the risk of IBD [10]. Smoking, in particular, is a significant risk factor for CD, with smokers having a higher risk compared with non-smokers. Quitting smoking can help reduce the risk of developing CD and may also improve the course of the disease [10]. Timely and accurate diagnosis is imperative for initiating appropriate treatment and managing disease progression. Early diagnosis is particularly crucial for enhancing treatment outcomes, mitigating or delaying the need for surgery, and improving patients’ quality of life. This review highlights the importance of an integrated diagnostic approach, including clinical evaluation, laboratory tests, imaging techniques, and endoscopy with biopsy, in the diagnosis of IBD (Table 1). A diagnostic algorithm for early detection of the disease is proposed (Figure 1).

## 2. Clinical Approach

In clinical practice, diagnosing IBD is complex because of the wide variety of gastrointestinal symptoms reported by patients, which are neither specific nor exclusive to IBD [2]. Furthermore, several other gastrointestinal disorders mimic the clinical presentation of IBD, making a differential diagnosis crucial [2]. According to the ECCO consensus, the main gastrointestinal symptoms of UC are visible blood in the stools, rectal urgency, and tenesmus, reported in over 95% of the active disease [11]. In contrast, chronic diarrhea is the most common onset symptom in ileocolonic CD, followed by abdominal pain and weight loss [12]. A significant percentage of patients (up to 20%) with Crohn’s disease present complications such as strictures or fistulas at the time of diagnosis [13]. Stricture disease is a recognized complication of CD and results from recurrent chronic inflammation [14]. The presence of symptoms of perianal disease should raise suspicion of CD, considering that up to 10% of newly diagnosed CD cases present with such involvement [13]. Furthermore, in patients with IBD, symptoms meeting the criteria for IBS, such as chronic abdominal pain associated with altered bowel habits, may be present. In patients with active inflammatory disease, the prevalence of symptoms compatible with IBS can reach 44% and are more common in patients with Crohn’s disease compared with those with ulcerative colitis [15]. An acute onset of symptoms, presence of abdominal pain, bloody diarrhea, and extraintestinal manifestations can strongly suggest the presence of IBD while alternating diarrhea and constipation are more strongly associated with functional disease rather than IBD [16]. Abdominal pain is a typical feature of IBD, and about 71% of patients report this symptom [17]. However, the presence of persistent abdominal pain should prompt physicians to consider other diagnoses beyond IBD, as it is rare in IBD except in cases of severe acute colitis [16]. Acute onset of symptoms with abdominal pain in the right iliac fossa and fever may occur in patients with Crohn’s disease confined to the appendix, mimicking the symptoms of acute appendicitis and leading the patient to emergency surgery. This entity is rare, accounting for 1.8% of all appendicitis patients undergoing emergency surgery [18]. The incidence of appendicular Crohn’s disease is variable and is generally described as 0.2–0.55%. Appendicular Crohn’s disease usually occurs in young people, and approximately 25% of patients have other symptoms associated with Crohn’s disease, such as chronic abdominal pain and chronic diarrhea [19]. According to ECCO statements, there are several clinical scoring systems presently available to classify disease severity in ulcerative colitis and Crohn’s disease. The Mayo Clinic Score is an easy composite assessment of clinical symptoms [stool frequency and rectal bleeding] and is the most used assessment in UC, while the most common clinical activity index in CD is the Harvey–Bradshaw Index [20] (Table 2 and Table 3).

The presence of extraintestinal manifestations (EIMs) associated with gastrointestinal symptoms should always be investigated [21]. The most common manifestations involve the musculoskeletal system (such as peripheral and axial arthritis), skin (including pyoderma gangrenosum, erythema nodosum), hepatobiliary tract, and eyes (episcleritis, anterior uveitis, and iritis) [21]. Finally, approximately 10–15% of IBD cases cannot be definitively classified as ulcerative colitis or Crohn’s disease [22]. The term “IBDU” (indeterminate inflammatory bowel disease) was used in 2005 to describe those cases where clinical presentation, endoscopic findings, and biopsy results suggest the presence of IBD, but a clear distinction between ulcerative colitis and Crohn’s disease cannot be made [23]. Several other gastrointestinal disorders can mimic the clinical presentation of IBD. Distinguishing these conditions from IBD initially involves a combination of clinical evaluation and laboratory tests. All patients with clinical suspicion of IBD should undergo stool examinations, including stool culture and testing for Clostridium difficile infection, to exclude enteric infections such as infectious colitis like Clostridium difficile infection, Salmonella, Shigella, Camphylobacter, Cytomegalovirus, or Yersinia enterocolitis. Other diseases that mimic IBD include gastrointestinal neoplasms and non-infectious enterocolitis such as ischemic colitis, diverticulitis, and irritable bowel syndrome [2]. In the diagnostic workup of inflammatory bowel disease, incorporating a detailed family history is crucial. Patients with a first-degree relative diagnosed with IBD have a higher likelihood of developing the disease themselves compared with those with no family history [24]. The presence of other immune-mediated diseases aids in the diagnosis, as such conditions may precede the clinical manifestations of IBD [25]. Recent research has highlighted the association between IBD and autoimmune pancreatitis (AIP). It has been observed that AIP may precede the diagnosis of IBD in a substantial percentage of cases (59% of patients), underscoring the importance of considering autoimmune conditions in the diagnostic process [25].

### Biomarkers

IBD is associated with several biochemical alterations as increases in inflammatory markers, including C-reactive protein (CRP), fecal calprotectin (f-cal) and lactoferrin, vitamin and mineral deficiencies, and anemia [2]. Fecal calprotectin, a protein detectable in stool samples that is correlated with increased neutrophils in the intestine, can be a valuable tool in the diagnostic process to rule out IBD [2]. Research suggests that patients with low levels of fecal calprotectin have less than a 1% chance of being diagnosed with IBD [26]. A meta-analysis review evaluated the diagnostic accuracy of fecal calprotectin for IBD. The authors found that the overall sensitivity of fecal calprotectin in detecting IBD was 85.8%, while the specificity was 91.7%, suggesting its ability to rule out IBD. The findings suggested that fecal calprotectin testing, particularly when using a** cut-off value of >50 μg/g, could be a useful non-invasive tool in the diagnostic workup of IBD [27]. A calprotectin level above 50 µg/g on two separate occasions must lead to further investigation such as colonoscopy and/or intestinal imaging to confirm IBD [28]. Other studies have shown that patients with higher levels (>250 µg/g) of calprotectin may be more suggestive of active intestinal inflammation. Patients with calprotectin levels exceeding 250 µg/g have an 8% chance of developing IBD within 1 year, compared with a 1% chance in those with levels below 50 µg/g [29].

However, it is known that calprotectin is not a biomarker specific to IBD. For example, f-cal concentrations are elevated during Salmonella infection (median of 765 µg/g), Campylobacter infection (median of 689 µg/g), and Clostridioides difficile infection (median of 740 µg/g) [30]. Additionally, elevated f-cal concentrations have also been reported in patients with HIV infection [31]. Lactoferrin is an iron-binding glycoprotein also found in neutrophil granules with antimicrobial properties [32]. It was reported that positive lactoferrin correlated with histological inflammation and C-reactive protein and was present in 92% of CD patients and 83.3% of UC patients with intestinal inflammation [32]. Serological markers, such as anti-Saccharomyces cerevisiae antibodies (ASCAs) and anti-neutrophil cytoplasmic antibodies (ANCAs), have limited value in making an initial definitive diagnosis of IBD. However, they may help clinicians distinguish between Crohn’s disease and ulcerative colitis, predict disease behavior, and determine prognosis [2,33]. Genetic testing for specific IBD-associated polymorphisms contributes to personalized risk stratification and therapeutic decision-making but is currently not recommended for the routine diagnosis of CD and UC [2,34].

## 3. Endoscopy

Endoscopy plays a fundamental role in the diagnosis of IBD [35]. It provides direct visualization of the gastrointestinal tract, allowing clinicians to assess the extent and severity of inflammation, identify characteristic features of IBD, and guide treatment decisions [35]. For patients with clinical presentations suggestive of IBD, colonoscopy with intubation of the terminal ileum is recommended as part of the diagnostic workup [36]. To ensure thorough assessment and accurate diagnosis, it is recommended to obtain at least two biopsies from six different areas during colonoscopy, including the terminal ileum, ascending colon, transverse colon, descending colon, sigmoid colon, and rectum [36]. The visualization of the ileum is essential for the differential diagnosis between ulcerative colitis and Crohn’s disease, especially in patients with symptoms and/or radiographic findings suggesting a diagnosis of CD. When ulcers or erosions are present in the ileum, biopsies are mandatory [37]. Furthermore, ileocolonoscopy was found to have an accuracy of 89% in differentiating UC from CD in a prospective study [38]. Classic endoscopic findings in ulcerative colitis include erythema, loss of normal vascularity, mucosal granularity, erosions, friability, bleeding, and ulcerations. The disease generally begins in the rectum, extending proximally in a continuous, circumferential pattern [39]. Skip and patchy lesions are not typically associated with ulcerative colitis (UC) and refer to areas of disease involvement that are separated by normal or unaffected tissue [36]. Recent evidence suggests that skip lesions, particularly appendiceal orifice inflammation (AOI), can occur as well with a prevalence from 7.9 to 75.0% in UC patients observed endoscopically [40]. Appendiceal orifice inflammation (AOI) refers to inflammation affecting the area around the opening of the appendix into the cecum and is considered a type of skip lesion in the context of UC [41]. Rectal sparing or patchy disease occurred in 32.1% and 30.4% of patients by endoscopy, and no correlation was noted among any specific type or combination of medication use [42]. Up to 20% of patients with colitis can have mild inflammatory changes in the terminal ileum called backwash ileitis (extension of the inflammatory process into the terminal ileum) [36]. The presence of backwash ileitis is not associated with greater disease severity. It is often linked with pancolitis, a significantly shorter disease duration (*p* < 0.005), and primary sclerosing cholangitis (*p* < 0.001) [43]. Endoscopic features suggestive of CD include aphthous ulcers, deep ulcers, serpiginous ulcers, cobblestoning, and stenosis [39]. Features that favor CD ileitis include extensive inflammation, patchy inflammation, and stricturing of the terminal ileum or ileocecal valve [44,45]. Esophagogastroduodenoscopy (EGD) should be considered in patients with symptoms suggestive of upper gastrointestinal (GI) tract involvement, which occur in up to 16% of patients with CD [45]. During EGD, at least two biopsies should be obtained from the esophagus, stomach, and duodenum to aid in diagnosis [36]. Endoscopic findings suggestive of upper GI CD may include aphthous ulcers, strictures, fistulas, and erythema [36]. These findings, along with histological examination of biopsy samples, can help confirm the diagnosis of CD and guide appropriate management strategies [36]. Endoscopic scores have been developed for UC and CD to assess disease severity. These systems should always be used in clinical decision-making. The most commonly used score for UC is the Mayo score. For CD, the most commonly used is the Simple Endoscopic Scoring System for Crohn’s Disease (SES-CD) [35] (Table 4 and Table 5).

## 4. Histological Diagnosis

According to ECCO’s position, histology can confirm the diagnosis of IBD, exclude dysplasia, exclude coexistent conditions or complications, and determine the severity and extension of disease (UC or CD) [46]. However, several diseases can histologically mimic inflammatory bowel disease. Correlating histological features with clinical and endoscopic findings is essential to facilitate accurate diagnosis [47]. In the evaluation of biopsies, three main categories may be considered as follows: crypt architectural, lamina propria cellularity, and epithelial abnormalities [46,48,49].

### 4.1. Crypt Architectural

Normal crypts in an oriented biopsy are parallel to each other and extend from the surface to the muscular mucosa. Biopsy orientation is essential because imperfect orientation can give a false impression of crypt distortion or shortening [50]. Crypt distortion in colonic mucosa is defined by the presence of non-parallel crypts, with variable or cystically dilated diameters compared to normal (minimum of 10%), or by increased crypt branching (if less than 10% of all crypts, it is not abnormal) and/or variation in crypt size and shape (reduced proportion of total colonic mucosa area occupied by crypts) [46]. Crypt shortening (atrophy) is an increase, usually variable, in the distance between the crypt bases and the muscular mucosa. Villous atrophy in ileal mucosa is defined as the presence of shortened villi with a villus-to-crypt ratio of less than 3:1 [46,48].

### 4.2. Lamina Propria Cellularity: Chronic and Acute Inflammation and Epithelioid Cell Granuloma

In the normal large intestine, the majority of cells are located in the upper third of the lamina propria with a ratio between the superficial and basal thirds that is nearly 2:1 [50]. The lamina propria harbors a diverse array of immune and non-immune cells, including lymphocytes, macrophages, fibroblasts endothelial cells, plasma cells, and granulocytes [51]. During inflammation, these resident cells undergo proliferation and recruitment, leading to an overall increase in cellular density within the lamina propria [52]. An increased number of plasma cells (basal plasmacytosis), lymphocytes, histiocytes, neutrophils, and eosinophils are characteristic of all types of colorectal inflammation and are not specific to IBD [46,48]. In IBD biopsies, chronic inflammation is defined as an increase in the number of lymphocytes and plasma cells in the lamina propria [46,48]. Eosinophils do not define chronic inflammation and are relatively rare compared with plasma cells and lymphocytes. There is an unknown variation in the site of intramucosal eosinophils (they are commonly present in the proximal colon and more present in samples obtained in April and May) that likely reflects different allergen exposure [52]. Acute inflammation is defined histologically by the presence of neutrophils, either in the lamina propria, crypt epithelium, or crypt lumens. Cryptitis is defined as the presence of at least one neutrophil in the crypt epithelium, while a crypt abscess is defined as the presence of more than one neutrophil in the crypt epithelium the lumen of the crypt [48,53]. Two main patterns of increase in the lamina’s cellularity are described to be diffuse and discontinuous [46,48,49]. Within diffuse increase, there are two subgroups including a diffuse superficial increase, which is limited to the surface of the lamina propria, and a diffuse transmucosal increase, resulting in a uniform transmucosal distribution of cells [46,48,49]. A discontinuous increase is a variable (focal or patchy) increase in lamina propria cellularity in the biopsy specimen and is not limited to the superficial zone [48,49]. An epithelioid cell granuloma is a collection of at least five epithelioid cells (activated histiocytes), accompanied or not by multinucleated giant cells [54,55]. In CD, a granuloma never has caseous necrosis and typically shows no confluence with other granulomas [54,55]. Epithelioid granulomas are not specific to IBD. However, they represent one of the best histological criteria for distinguishing Crohn’s disease (CD) from UC. Among patients with CD, granulomas are documented in biopsy samples in only 12% of cases [56]. 

### 4.3. Epithelial Abnormalities

According to ECCO’s definitions, the epithelial abnormalities in IBD are mucin depletion, i.e., a reduction in the number of goblet cells or a reduction in mucin droplets. Pyloric metaplasia is the replacement of the original epithelium with glands resembling those in the gastric antrum and surface epithelial damage, e.g., focal cell loss, flattening, erosions, and ulcers that reflect the activity of disease [46,48,49].

In conclusion, the most frequent alterations in IBD include abnormal crypt architecture, basal plasmacytosis, increased cellularity of the lamina propria, mucin depletion, granulomas, crypt abscesses, cryptitis, and ulcerations [55,57,58]. While in infectious colitis, it is common to observe acute inflammatory changes such as cryptitis, crypt abscesses, lamina propria neutrophils, and edema. Basal plasmacytosis is the strongest predictor of IBD [57,58,59,60] (Table 6).

## 5. New Advance Endoscopic Techniques

“Dye-chromoendoscopy” is a technique used during endoscopic examinations to improve visualization of the gastrointestinal tract mucosa [61]. This method involves applying different dyes directly onto the mucosa, which highlights subtle changes in tissue structures and aids in the detection of abnormalities such as dysplasia, polyps, or early-stage cancers. Endoscopists typically use dyes like indigo carmine or methylene blue to enhance visualization [61]. In a recent study, chromoendoscopy was associated with a higher dysplasia detection rate than white-light endoscopy in UC patients and helped to predict neoplastic changes during the endoscopic procedure [62]. Virtual chromoendoscopy (VCE) is widely used in endoscopy to enhance the details of mucosal and vascular patterns without dye application [63,64]. VCE includes various optical technologies such as narrow-band imaging (NBI; Olympus), flexible imaging color enhancement (FICE; Fujinon), and blue laser light/linked color imaging (BLI/LCI; Fujifilm) [63]. These techniques use optical filters and/or digital processing to improve the visualization of mucosa and vascular structures without requiring the application of dyes [64]. This advanced imaging technology represents an alternative to traditional chromoendoscopy, offering detailed images of the gastrointestinal mucosa. NBI enhances contrast by illuminating the mucosa with specific wavelengths of light, highlighting surface patterns and vascular structures [64,65]. This improved visualization aids in the detection and characterization of dysplasia in UC [65]. FICE and iSCAN are based on the same physical principle as NBI, but because of a computed spectral estimation technology, they are not dependent on the presence of optical filters inside the video endoscope [66,67,68]. Neuman et al. demonstrated that iSCAN (Pentax) could specifically predict, without histological examination, the extent of inflammation in 92.31% of patients and disease activity in 89.74% of patients with IBD [69]. Additionally, new endoscopic devices allow real-time in vivo histology during endoscopic examination [66,70]. Confocal laser endomicroscopy (CLE) is a diagnostic imaging technique used during endoscopic procedures to obtain real-time, high-resolution images of the gastrointestinal mucosa at the cellular level. CLE allows real-time in vivo *histology* imaging at approximately 1000 times magnification and approximately 1 micron resolution [63]. This technique involves illuminating the mucosal surface with a low-power laser and collecting the reflected fluorescent light. CLE relies on tissue fluorescence for imaging; therefore, the use of an intravenous contrast agent, such as fluorescein (1.0–5.0 mL 10%), is generally required. Fluorescein increases tissue contrast and improves the visualization of cellular structures, aiding in the identification and characterization of abnormalities in the gastrointestinal mucosa [63]. Several studies have explored the utility of endomicroscopy in the in vivo diagnosis of mucosal changes associated with inflammatory bowel disease [70,71,72]. In particular, comparative studies have been conducted between images obtained through confocal endomicroscopy and histological images of biopsy samples obtained during colonoscopy in patients with ulcerative colitis. These studies demonstrate that images captured with confocal microendoscopy can provide information equivalent to conventional histology [70,71,72]. Confocal laser endomicroscopy has demonstrated the capability to detect microscopic inflammatory changes in a macroscopically non-inflamed, normal-appearing mucosal pattern [73]. Additionally, a new classification of inflammatory activity in ulcerative colitis using endomicroscopy has been proposed, which includes assessments of crypt architecture and microvascular alterations [74]. In that study, endomicroscopy was found to be reliable for real-time assessment of inflammatory activity in ulcerative colitis, with good correlations observed with histological findings (Spearman’s rho, both *p* < 0.001) [74]. Furthermore, CLE can play a significant role in IBDU as a valuable tool in the diagnostic algorithm. Hundorfean et al. proposed CLE-based criteria to help in this differential diagnosis. According to their findings, characteristic features observed with CLE in CD include mucosal fissures, focal cryptitis, granulomas, and microscopic inflammation of the terminal ileum. In contrast, patients with UC often exhibit characteristic bifid, shortened, and branched crypts, along with microscopically normal terminal ileum [75].

## 6. Wireless Video Capsule Endoscopy and Enteroscopy

Wireless video capsule endoscopy (VCE), first approved in 2000, was developed to allow non-invasive visualization of the entire small intestine [76]. It has been observed that up to 30% of the patients with Crohn’s disease have only small bowel involvement, without involvement of the terminal ileum; in these patients, traditional endoscopic examinations do not allow for disease diagnosis [77]. The European Society of Gastrointestinal Endoscopy (ESGE) recommends VCE as the initial diagnostic modality to investigate the small bowel in patients with suspected Crohn’s disease and negative findings on ileocolonoscopy, provided there are no obstructive symptoms or known bowel stenosis [78]. The non-invasive nature of VCE represents a significant advantage due to its ability to detect early mucosal lesions with higher sensitivity than conventional radiology [79,80]. Petruziello et al. demonstrated that VCE is particularly useful in patients with suspected CD who have previously undergone endoscopic and radiological negative examinations, being able to detect lesions compatible with CD in the small intestine in almost a third of patients [81]. VCE also documented proximal lesions of the small intestine in patients with known Crohn’s disease in a high percentage of cases (up to 50%), especially in those with Crohn’s disease localized to the terminal ileum [82]. Typical VCE findings of CD include erythema, erosions, ulcerations, and strictures [80]. VCE examination leads to a risk of capsule retention, especially in patients with suspected strictures. Capsule retention has been reported in up to 13% of patients undergoing capsule studies for CD [83]. However, the development of patency capsules has reduced the risk of capsule retention in patients with known or suspected strictures [82]. Given the high diagnostic sensitivity of less invasive tests for studying the small intestine, device-assisted enteroscopy (DAE) has a limited role in the initial evaluation of patients with suspected IBD [84]. DAE is a generic term for the assisted progression of an enteroscope into the small bowel [85]. The assistance is provided by overtubes, balloon catheters, or other stiffening devices. Currently, the available systems are enteroscopy with a semi-rigid overtube, double-balloon enteroscopy, single-balloon enteroscopy, and spiral enteroscopy [85]. DAE may be preferable to VCE if there is a clinical suspicion of obstruction because it allows for therapeutic intervention, avoiding capsule retention, or if there is a strong clinical suspicion of CD but previous radiological, endoscopic, and VCE investigations are inconclusive [84]. The disadvantages of DAE include the invasiveness of the exam, the need for sedation, occasional difficulty in exploring the entire small intestine, and the time and costs involved. However, its advantages include the ability to perform biopsies and directly visualize atypical lesions [84]. *Heine* et al. showed that enteroscopy was able to diagnose CD of the small intestine in up to 30% of cases [86]. Furthermore, in patients with a high clinical suspicion of CD presenting with chronic diarrhea, abdominal pain, perianal lesions, and systemic symptoms, the diagnostic accuracy of DAE in diagnosing CD is approximately 60% [87].

## 7. Advanced Imaging Techniques

Advanced imaging techniques, including computed tomography enterography (CTE), magnetic resonance enterography (MRE), and intestinal ultrasound (IUS), provide complementary information useful in the diagnosis of IBD by contributing to the assessment of the extent of small bowel involvement in Crohn’s disease, evaluating the presence of complications, and assessing disease activity [88,89]. According to ECCO and ESGAR (European Society of Gastrointestinal and Abdominal Radiology) guidelines, all patients with suspected and/or symptomatic small bowel disease can be investigated with radiological imaging techniques. The choice of the first investigation is based often on local availability and expertise [89]. Each examination should specify the number and anatomical location of inflamed intestinal segments, including skip lesions, specifying the total length of the segments affected by inflammation [90]. Parameters used to define inflammation include bowel wall thickness (BWT) (with a cut-off for the presence of mural inflammation both for the small and large intestine > 3 mm) [90,91]; mural changes such as ulcerations and edema [91,92]; increased intestinal vascularity, reflecting neoangiogenesis in inflamed tissue [93]; and finally, the presence and location of perienteric inflammatory alterations such as mesenteric adipocyte proliferation [94]. MRE has become a widely accepted method for conducting a detailed assessment of the small intestine in patients with Crohn’s disease. The METRIC study, a multicenter study that compared the diagnostic accuracy of MRE and ultrasound for the presence, extent, and activity of small bowel Crohn’s disease in newly diagnosed patients, showed an MRE sensitivity of 80% for assessing the extent and 97% for the presence of small bowel disease. The specificity of MRE for evaluating the extent of small bowel disease was approximately 95% [95]. Several indices have been developed to assess luminal activity in MRE. The most widely used MRE activity index [MaRIA] is a composite index that considers bowel wall thickness, quantifies enhancement in the bowel after gadolinium injection, and identifies ulceration and bowel edema [20]. Recently, another index, the Simplified Magnetic Resonance Activity Index (MARIAs), has also been developed. MARIAs can be calculated without enhancement and has shown strong correlations with SES-CD [96]. Intestinal ultrasound has emerged as a valuable tool for small bowel evaluation, offering several advantages over other imaging techniques. IUS is a non-invasive procedure that does not involve needles or radiation exposure, assists in real-time clinical decisions, is cost-effective, requires no oral preparation, and is easily reproducible. This makes it a safer and more comfortable experience for patients compared with CTE and MRE [20,97,98]. IUS can effectively visualize significant portions of the bowel such as most of the large intestine and major parts of the small intestine, with the exception of the proximal jejunum. For optimal visualization of the bowel wall layers, which are typically less than 3 mm thick, a transducer frequency of at least 5 MHz is necessary [20]. The diagnostic accuracy of IUS was 91% for the localization of disease, with a sensitivity of 92% [95,99]. In CEUS (contrast-enhancement ultrasound), the uses of intravenous contrast provide a more quantitative assessment of mural and extramural vascularity [98]. Its routine use for Crohn’s disease activity assessment is not yet established, and the IUS remains the preferred method because of its practicality and established role in clinical practice. However, CEUS may play a future role in specific situations like differentiating inflammation from fibrosis [98]. CTE of the abdomen and pelvis with luminal distension and intravenous contrast is another valuable tool for evaluating IBD, particularly Crohn’s disease. It provides detailed information about bowel wall involvement, complications like strictures and fistulas, and mesenteric vessel abnormalities [89]. However, the potential for radiation exposure necessitates a careful evaluation of risks and benefits before performing a CT scan. Its ability to rapidly assess the entire gastrointestinal tract and guide interventional procedures makes it invaluable for patients especially in the presence of complicated disease [89]. Recent advances in medical imaging have also included the use of several techniques to evaluate fibrosis. We know that the presence of stenosis disease is associated with a 4–5-fold higher need for surgical resection over time in CD [100]. So, the early diagnosis of fibrosis allows for the prediction of disease progression risk and the need for intervention [100]. These techniques encompass novel cross-sectional imaging modalities like positron emission tomography (PET), specific magnetic resonance imaging (MRI) sequences such as diffusion-weighted imaging (DWI), an advanced sequence on magnetic resonance imaging that maps the diffusion of water molecules in biological tissues, and ultrasound-based elastography technologies, a non-invasive ultrasonographic tool to detect the tissue stiffness of stenotic bowel. The strain rate is a measure of the rate of deformation and so a measure of fibrosis [101,102]. Some authors have developed a hybrid biomarker using three enterographic/PET biomarkers, showing significant differences in the fibrosis group compared with the group with active fibrosis and inflammation [103]. *Foti* et al. instead demonstrated, through a combination of parameters derived from conventional MR sequences and DWI, that a restricted diffusion pattern detected by DWI in the intestinal wall is associated with the presence of active inflammation and not fibrosis [104]. Fraquelli et al. documented elevated strain elastography ratio values for patients with fibrotic strictures compared with those with moderate fibrosis or inflammation [105]. Radiological imaging techniques offer valuable tools for the diagnosis of IBD. The choice of imaging modality depends on the specific clinical question, patient factors, and availability of resources. Balancing the advantages of detailed visualization and diagnostic accuracy with the potential drawbacks of radiation exposure, contrast agent administration, and costs is crucial for optimizing patient care (Table 7).

## 8. Future Directions—Pre-Clinical Diagnosis of Inflammatory Bowel Disease

Early clinical diagnosis is always to be sought, but several studies indicate that a significant number of patients experience gastrointestinal symptoms more than 6 months before receiving a diagnosis [106]. These symptoms may correlate with abnormalities in biological tests such as elevated C-reactive protein (CRP), leukocytes, increased fecal calprotectin, abnormalities in alanine aminotransferase, or decreased levels of hemoglobin, iron, folate, or vitamins D2 and D3 alterations [106]. This preclinical phase of IBD is often attributed to delayed diagnosis [106]. Vestergaard et al. observed the most significant differences in laboratory tests in the year immediately preceding the diagnosis. Most of the detected abnormalities (excluding fecal calprotectin) did not exceed normal test values, likely explaining why they are not further investigated [107]. This evidence has prompted the development of risk models to predict the likelihood of developing IBD. Such predictive tools could facilitate early diagnosis in asymptomatic patients [108]. In order to minimize diagnostic delays, Danese et al. developed a tool to identify early CD. The “Red Flags questionnaire” is a tool comprising relevant signs and symptoms suggestive of IBD, such as nocturnal diarrhea, the presence of complex perianal fistula or abscess, weight loss, chronic abdominal pain, the absence of rectal urgency, positive family history of IBD, and mild fever in the last 3 months. Subjects with a Red Flags score ≥ 8 were significantly more likely to have Crohn’s disease. These patients underwent laboratory tests including blood cell count, serum C-reactive protein, and fecal calprotectin (FC), and were subjected to IUS. The Red Flags questionnaire combined with elevated fecal calprotectin (FC > 250 ng/g) significantly improved the diagnostic accuracy of CD with a sensitivity of 100% and a specificity of 72% [109,110].

## 9. Discussion

The integration of evidence from multiple approaches allows for early diagnosis, improves diagnostic accuracy, and facilitates differentiation between CD and UC. Histological examination, along with endoscopic, clinical, and imaging data, confirms the diagnosis of IBD or helps to exclude it. The duration of diagnostic delay is associated with an increased risk of intestinal stenosis and intestinal surgery in Crohn’s disease [111,112]. Several studies confirm that there is a preclinical phase that begins much earlier than previously thought, especially in Crohn’s disease. In the future, we should develop strategies to identify individuals at risk of developing the disease before it manifests, addressing risk factors such as smoking, diet, and environmental factors. Imaging acquired through MRE, CTE, and IUS is valuable for diagnosis, particularly in patients with small intestine involvement, but it does not confirm chronic inflammatory disease. For patients with clinical suspicion of Crohn’s disease, it is recommended to initially perform an abdominal ultrasound because of its non-invasiveness and immediate feasibility [113,114,115]. Histological examination plays a crucial role in undefined cases of IBD, as it can definitively exclude or confirm the diagnosis [58,59]. The potential of confocal laser endomicroscopy to evaluate inflammation without requiring biopsies holds promise and could improve the efficiency and accuracy of assessing IBD [69,71,73]. Further studies are necessary to validate CLE’s capability to assess inflammation thoroughly. However, there are currently no studies on the use of CLE in patients without a prior diagnosis of IBD, and the role of CLE in determining the actual subtype of IBDU has yet to be determined. Ultimately, within the diagnostic algorithm, the gastroenterologist plays a fundamental role in determining the extent and severity of the disease through endoscopic examination, confirming or excluding small intestine involvement through VCE and IUS, and, potentially, with the advent of CLE, confirming the histological diagnosis of IBD.

## Figures and Tables

**Table 1:** The combination of clinical, laboratory, and diagnostic tools helps the clinician in the diagnosis of IBD. Inflammatory bowel disease: IBD, Crohn’s disease: CD, device-assisted enteroscopy: DAE, video capsule endoscopy: VCE, intestinal ultrasound: IUS, confocal laser endomicroscopy: CLE, magnetic resonance enterography: MRE, CTE: computed tomography enterography.

Tools for Diagnosis of IBD	
Clinical evaluation	Evaluate presence of gastrointestinal symptomsa and Extra intestinal symptoms; family history of IBD; autoimmune disease
Laboratory tests	Stool examinations for enteric infections; fecal calprotectin and lactoferrin test; laboratory tests such as C-reactive protein, blood cell count, iron, and vitamins
Endoscopy	Ileocolonscopy with biopsies: in all patients with suspected IBD; Esophagogastroduodenoscopy with biopsies: if upper symptoms; VCE: if suspected CD and negative findings on ileocolonoscopy; DAE: to take biopsies or when stenosis is expected; CLE: for inflammation assessment
Imaging techniques	IUS, MRE, or CTE to evaluate the extent of small bowel involvement in CD, disease activity, and complications; IUS and CTE to evaluate the extent of disease and complications in ulcerative colitis

**Table 2:** **The partial Mayo score for ulcerative colitis.** This score combines two patient-reported outcomes (stool frequency and rectal bleeding) and the physician’s global assessment and defines the activity disease. The Mayo score ranges from 0 to 12, with higher scores indicating more severe disease.

Partial Mayo Score [Index]	0	1	2	3
**Stool frequency**	Normal	1–2/day > normal	3–4/day > normal	5/day > normal
**Rectal bleeding**	None	Streaks	Obvious	Mostly blood
**Physician’s global assessment**	Normal	Mild	Moderate	Severe

**Table 3:** **Harvey–Bradshaw Index (HBI) for assessing Crohn’s disease activity**. The HBI is a simple scoring system for assessing the degree of illness in patients with Crohn’s disease. The index considers five clinical parameters, and for each parameter, a specific score is assigned. The HBI evaluates general well-being and abdominal pain the day before; the number of liquid or soft stools (previous day); the presence of abdominal mass; and the presence of complications.

Variable	Variable Description
**General well-being**	0 = very well; 1 = slightly below average; 2 = poor; 3 = very poor; 4 = terrible
**Abdominal pain**	0 = none; 1 = mild; 2 = moderate; 3 = severe
**Number of liquid stools**	0 = 0–1 stools; 1 = 2–3 stools; 2 = 4–5 stools; 3 = 6–7 stools; 4 = 8–9 stools; 5 = 10+ stools
**Abdominal mass**	0 = none; 1 = dubious; 2 = definite; 3 = tender
**Complications**	None, uveitis, arthalgia, erythema nodosum, aphthous gangrenosum, anal fissure, new fistula, abscess; one point each

The total HBI score is calculated by summing the individual scores for each parameter. Remission < 5; mild disease 5–7; moderate disease 8–16; severe disease >16.

**Table 4:** Simple endoscopic scoring system for CD activity (SES-CD). The SES-CD is a simple and reproducible endoscopic scoring system. It considers the following 4 endoscopic variables: the presence and the size of ulcers, the proportion of the surface covered by ulcers, the proportion of the surface with any other lesions, and stenosis. Each variable was scored from 0 to 3 in each segment (the ileum; the right colon segment; the transverse colon; the left colon segment and the rectum segment). The score is correlated to clinical and biochemical parameters such as serum C-reactive protein level.

Variable	0	1	2	3
Size of ulcers	None	Afthous ulcers (Diameter 0.1 to 0.5 cm)	Large ulcers (Diameter 0.5 to 2 cm)	Very large ulcers (Diameter > 2 cm)
Ulcerated surface	None	<10%	10–30%	>30%
Affected surface	Unaffected segment	<50%	50–75%	>75%
Presence of narrowings	None	Single, can be passed	Multiple, can be passed	Cannot be passed

**Table 5:** Mayo endoscopic score for UC disease activity. It consists of a 4-point scale from inactive (score 0) to severely active disease (score 3) based on endoscopic findings, such as erythema, vascular pattern, friability, bleeding, erosions, and ulcerations, evaluated in the most inflamed colonic area.

Mayo Endoscopic Score	Endoscopic Features
0	None
1	Erythema, decreased vascular pattern, mild friability
2	Marked erythema, absent vascular pattern, friability, erosions
3	Spontaneous bleeding, ulcerations

**Table 6:** Histological characteristics of ulcerative colitis, Crohn’s disease, and infectious colitis.

Typical Histological Features	Ulceratis Colitis	Crohn’s Disease	Infectious Colitis
Lymphoid aggregates	Frequent in mucosa	Common, transmural	Present in mucosa
Granulomas	Absent	Common, transmural	Possible in tubercolosis enteritis
Localization of inflammation	Limited to the mucosa	Transmural	Limited to the mucosa
Active inflammation	Diffuse	Focal with skip lesions	Diffuse
Cryptitis, crypt abscesses	Diffuse continuous	Focal discontinuous	Frequent, diffuse
Crypt architectural distortion	Diffuse	Focal, frequent	Usually absent
Atrophy	Present	Uncommon, mild	Rare
Pyloric metaplasia	Rare	Present	Rare except in tuberculosis enteritis
Basal plasmacytosis	Present	Present	Usually absent

**Table 7:** Summary of the advantages and disadvantages of the main radiological techniques.

Radiological Imaging Techniques in Inflammatory Bowel Disease	Advantages of Radiological Imaging Techniques in IBD	Disadvantages of Radiological Imaging Techniques in IBD
Magnetic resonance enterography	Non-invasive nature Assessment of disease extent and severity Evaluation of disease complications Sensitivity > 97%	Radiation exposure Contrast agent administration High cost Contraindicated in patients with certain medical devices or claustrophobia Diagnostic accuracy for stenosis is based on the use of luminal contrast
Intestinal ultrasound	Non-invasive nature Assessment of disease extent and severity Cost-effective No oral preparation Easily reproducible Evaluation of disease complications Sensitivity > 92%	Operator dependence Poor image quality in patients with excess abdominal weight
Computed tomography enterography	Non-invasive nature Assessment of disease extent and severity Evaluation of disease complications Sensitivity > 84%	Radiation exposure Contrast agent administration High cost

## Data Availability

All data generated or analyzed in this study are included in this article. Further inquiries can be directed to the corresponding author.
